# Artificial Intelligence to Improve Risk Prediction with Nuclear Cardiac Studies

**DOI:** 10.1007/s11886-022-01649-w

**Published:** 2022-02-16

**Authors:** Luis Eduardo Juarez-Orozco, Riku Klén, Mikael Niemi, Bram Ruijsink, Gustavo Daquarti, Rene van Es, Jan-Walter Benjamins, Ming Wai Yeung, Pim van der Harst, Juhani Knuuti

**Affiliations:** 1 Department of Cardiology, Division Heart & Lungs, University Medical Center Utrecht, Utrecht University, Utrecht, the Netherlands; 2 Turku PET Centre, University of Turku and Turku University Hospital, Kiinamyllynkatu 4-8, 20520 Turku, Finland; 3 Department of Cardiology, University of Groningen, University Medical Center Groningen, Groningen, The Netherlands; 4 Division of Imaging Sciences and Biomedical Engineering, King’s College London, St Thomas’ Hospital, London, UK; 5 Department of Artificial Intelligence, UMA-Health, Buenos Aires, Argentina

**Keywords:** Nuclear cardiology, Artificial intelligence, Risk prediction, Deep learning

## Abstract

**Purpose of Review:**

As machine learning-based artificial intelligence (AI) continues to revolutionize the way in which we analyze data, the field of nuclear cardiology provides fertile ground for the implementation of these complex analytics. This review summarizes and discusses the principles regarding nuclear cardiology techniques and AI, and the current evidence regarding its performance and contribution to the improvement of risk prediction in cardiovascular disease.

**Recent Findings and Summary:**

There is a growing body of evidence on the experimentation with and implementation of machine learning-based AI on nuclear cardiology studies both concerning SPECT and PET technology for the improvement of risk-of-disease (classification of disease) and risk-of-events (prediction of adverse events) estimations. These publications still report objective divergence in methods either utilizing statistical machine learning approaches or deep learning with varying architectures, dataset sizes, and performance. Recent efforts have been placed into bringing standardization and quality to the experimentation and application of machine learning-based AI in cardiovascular imaging to generate standards in data harmonization and analysis through AI. Machine learning-based AI offers the possibility to improve risk evaluation in cardiovascular disease through its implementation on cardiac nuclear studies.

**Graphical Abstract:**

AI in improving risk evaluation in nuclear cardiology. * Based on the 2019 ESC guidelines

**Figure:**
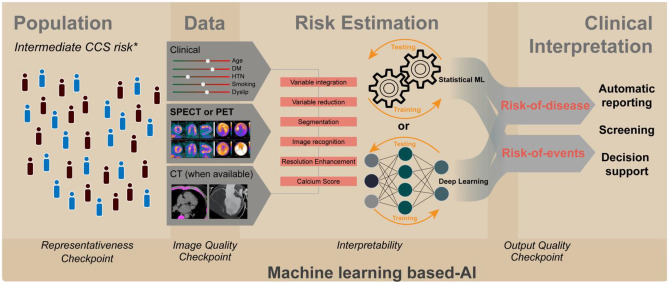

## Introduction

As machine learning-based artificial intelligence (AI) continues to revolutionize the way in which we analyze data, the field of nuclear cardiology provides fertile ground for the implementation of these complex analytics in the continuous search for optimizing the evaluation of known or suspected cardiovascular disease, mainly in the form of coronary artery disease (CAD) [1]. The flagship techniques of single photon emission computed tomography (SPECT) and positron emission tomography (PET) and their now almost standard hybridization with coronary computed tomography angiography (CCTA) provide a very large amount of data with both simple and complex patterns that continue to be harnessed by machine learning either its statistical form or through deep learning.

Given the increasing reports and recommendations regarding the experimentation with and implementation of machine learning-based AI in nuclear cardiology [2, 3], the present review sought to summarize and discuss the principles regarding nuclear cardiology techniques and AI, and the current evidence regarding its performance and contribution to the improvement of risk prediction in cardiovascular disease either by means of probability of disease estimation or longitudinal evaluation for the development of major adverse cardiovascular events.

## Nuclear Cardiac Studies (Nuclear Cardiology)

Although nuclear imaging is a powerful and versatile approach to target a variety of (patho)physiological processes to form a functional point of view (ranging from glucose consumption to innervation), its application in cardiology is dominated by myocardial perfusion assessment. A snapshot of the nature of the techniques, prevailing tracer, and demonstrated clinical value is presented ahead. Of note, both types of imaging are additionally able to deliver ventricular function proxies such as motion analysis, ejection fraction, and even synchrony through gated acquisition and reconstruction.

### Single Photon-Emission Computed Tomography

SPECT represents the most commonly used technique for assessing myocardial perfusion known or suspected CAD. The most recent hardware makes use of solid-state cadmium zinc telluride (CZT) detectors that directly detect single emitted 80–140 keV gamma rays (photons) from the injected and redistributed radiotracer (mainly ^99m^Tc-sestamibi or ^99m^Tc-tetrofosmin) in the patient’s heart. Recently, detectors are being integrated into novel collimator designs that increase photon sensitivity in the myocardial region; scanners are being reduced in size to facilitate implementation with high-throughput centers, and acquisition/processing protocols and software optimized to approach discrete PET performance (lower acquisition time, radiation exposure with higher resolution, and potential for quantification) with promising results [4].

The pooled performance of SPECT myocardial perfusion for the evaluation of myocardial ischemia resulting from anatomically obstructive CAD as in [5] is represented in Table 1.

**Table 1:** The performance of SPECT and PET for anatomically and functionally significant CAD ( Modified from: Knuuti et al. Eur Heart J Eur Heart J. 2018 Sep 14;39(35):3322–30, by permission of Oxford University Press) [5]

	**Sensitivity**	**Specificity**	**Positive LR**	**Negative LR**
*Anatomically significant CAD*				
**SPECT**	87 (83, 90)	70 (63, 76)	2.88 (2.33, 3.56)	0.19 (0.15, 0.24)
**PET**	90 (78, 96)	85 (78, 90)	5.87 (3.40, 10.15)	0.12 (0.05, 0.29)
*Functionally significant CAD*				
**SPECT**	73 (62, 82)	83 (71, 90)	4.21 (2.62, 6.76)	0.33 (0.24, 0.46)
**PET**	89 (82, 93)	85 (81, 88)	6.04 (4.29, 8.51)	0.13 (0.08, 0.22)

In terms of prognosis, the most recent long-term estimates for the occurrence of major adverse cardiovascular events (MACE) have emerged from the REgistry of Fast Myocardial Perfusion Imaging with NExt generation (REFINE) registry. In this study evaluating all-cause mortality, nonfatal myocardial infarction, unstable angina, or late coronary revascularization at roughly 5 years, a significant increase in MACE with worsening of visually assessed perfusion deficits, namely from 2.0% in normal to 7.4% in abnormal scans (while for semi-quantification through total perfusion deficit (TPD) values of 1.3% and 7.8% were respectively found for a TPD of 0% and > 10%) [6].

### Positron Emission Tomography

On the other hand, PET post-dates SPECT technology and represents the modern reference standard for quantification in nuclear imaging and thus, for the absolute quantification of myocardial blood flow. PET works through detection of photon pairs which are emitted from the injected radiotracer (^82^Rubidium, ^13^ N-ammonia, or ^15^O-water). A crucial difference in this detection is that by optimizing for pairs of photons and not for single photons, which is known as coincidence, less noise is recorded and more effective and organ located emissions can be detected. In conjunction, these characteristics yield lower radiation burden, higher imaging quality, and greater versatility in clinical acquisition logistics. Recently, fully digital PET/CT scanners are being deployed and are expected to boost current performance.

The pooled performance of PET myocardial perfusion for the evaluation of myocardial ischemia resulting from anatomically obstructive CAD has been introduced in [5] and depict in Table 1.

Prognostically, absolute quantitative perfusion with PET is independently associated with a range of MACE (i.e., cardiac death, acute coronary syndromes, revascularization, heart failure, stroke, and even peripheral vascular disease) with hazard ratios in the spectrum of 1.19–2.93] [7, 8].

### Hybrid Expansion of Nuclear Imaging Through Computed Tomography

Complementarily, an important proportion of nuclear studies is currently performed in conjunction with CT. In its simplest form, this provides a low-dose CT scan that serves as topographic base for the necessary corrections in the acquisition/processing of SPECT and PET, especially at the quantitative standard. Alternatively, a full CCTA can be performed prior to the nuclear acquisition either as gate-keeper (selective hybrid) for the nuclear study or as standard support in the same diagnostic step (standard hybrid). In low-dose images, apparent low image quality hinders diagnostic statements for obstructive CAD. However, modern approaches through machine learning (vide infra) open the possibility to extract sensitive and possibly diagnostically useful information from such coarse images. On the other hand, high-quality contrast images strongly supplement diagnostic considerations (with high sensitivity and excellent negative predictive value [5]) and also present a substrate for novel analytics [9].

## Machine Learning-Based Artificial Intelligence

Machine learning represents the family of algorithms (models) whose principal feature is the capacity to improve performance through iterative exposure to data (so-called training). Performance is measured with respect to the variable of interest that is set as outcome (“dependent variable” in conventional statistical terms) and the *ground truth* assumption around it. There is a wide variety of machine learning algorithms with varying rationales (e.g., k-nearest neighbor for clustering, random trees for decision rules, ensemble boosting for merging approaches in order to maximize performance, and deep artificial neural networks for text and image recognition [vide infra]). Artificial intelligence refers to the theory, generation, and study of systems/algorithms that are capable of performing applied tasks at an at least human intelligence level. Given the large strides achieved in parallel computing power and data storage, the modern AI era is grounded on machine learning algorithms, hence the term *machine learning-based AI*. In the context of nuclear cardiology, it means the chance to improve image acquisition, reduce radiation burden, improve image resolution, and clinically, boost the identification of disease in at-risk patients and the prediction of cardiovascular outcomes from the analysis and integration of medical imaging and/or clinical data.

In short, the process starts with data operationalization. This is then used as input to the algorithm in a parcellated structure that distinguishes training/validation data from test data. The former is used in iterations of training with intrinsic overfitting control through for example cross-validation in order to learn and select relevant features or patterns useful for completing the segmentation, classification, or prediction task. The latter is then used to evaluate the trained model’s performance in an independent dataset. Ideally, the test dataset may arise from a completely different cohort (true external validation). However, most often it is the original cohort that is divided into the model building and holdout test datasets.

Traditional machine learning methods (*statistical machine learning*), such as logistic regression, principal component analysis (PCA), random forest, support vector machine, boosting, and lasso regression, are used for classification from numerical and structured datasets (in the form of spreadsheets). Out of these, PCA exemplifies unsupervised methods, which are trained without known categories or outcomes. This is useful when exploring unknown subgroups or categories within a population or disease. In nuclear cardiac research, however, supervised methods have been predominantly employed.

### Deep Learning

In its simplest form, artificial neural networks consist of an input (structured, imaging, sound data), at least one intermediate, and an output layer (pseudoprobability of a category, regression or prediction). Additional intermediate layers are called hidden layers, and the more layers, the deeper the network becomes (as such now colloquially known as deep learning). During the last decade, the convolutional operator in the intermediate layers has become the gold standard in image segmentation and classification. Deep learning elucidates complex non-linear and high-dimensional patterns better than other machine learning strategies. Consequently, it has become the state-of-the-art for image (computer vision) and language (natural language processing) recognition tasks [10]. In essence, convolutions allow to stepwise flatten a 2- or 3-dimensional image to an array concerning its most relevant patterns. For example, before deep learning existed, convolutions were used for edge detection in image analysis. In training, neural networks are typically trained by in multiple epochs (whole runs of training data through a network) that allow the network to adjust its weights (coefficients) and select features that minimize a predefined loss-function (the penalties the network receives for incorrect predictions). This method has shown excellent performance, allowing cardiovascular researchers to deploy it in several forms using myocardial perfusion imaging data [11, 12]. Since training hyperparameters of these huge models from scratch could require massive amounts of data and computer resources, the possibility to exploit common characteristics of data (for example images) in pre-trained models for tasks that are broadly similar (picture classification vs. medical image classification) allows rapid adjustment of earlier networks to specific new tasks. The process of training a network on other similar data and then further fine-tuning it on actual data of interest is called transfer learning.

In the current standard for segmentation, a particularly successful image segmentation architecture is the “U-net.” This network exploits the information obtained during stepwise flattening (contracting path) in a stepwise up-sampling of the convolutional layers (expansive path) in order to propagate context information from the original image into the segmentation output image (https://arxiv.org/abs/1505.04597).

## The Evaluation of Risk in Cardiovascular Disease

The concept of risk in cardiovascular disease directly links to the process of probability estimation in health sciences. Although risk is conventionally employed to indicate prognostic estimations derived from longitudinal survival analysis, we believe that even punctual estimations of probability of an underlying disease (e.g., CAD) are to be considered within such concept given their morbid and therapeutic implications.

The natural history of CAD (being the most prevalent of cardiovascular diseases) presents the need to identify and monitor risk factors that contribute to its development, to promptly diagnose its presence and severity, and to characterize its expected progression or complication in order to implement interventions that modify it. Ergo, we permanently operate within the risk of developing CAD, of having undiagnosed CAD, and of presenting adverse CAD-related events. Notably, the extensive variability of complex biological systems presents a peculiar challenge in the estimation of probabilities regarding the disease’s clinical horizon at the level of the individual patient, one that can be approached through novel machine learning-based analytics.

In the following section, we summarize recent relevant studies in nuclear cardiology concerning the implementation of machine learning-based AI for the identification of CAD (risk of disease) and the prediction of CAD-related events (risk of MACE).

## AI Risk Prediction in Nuclear Cardiolgy

The study form 2017 by Motwani et al. [13] hallmarked the utilization of machine learning (through boosted ensembles) on cardiovascular imaging to retrospectively identify mortality at a 5-year mark. By combining clinical and coronary CT imaging data, the machine learning approach demonstrated a significantly improved identification of patients with mortality due to any cause (AUC = 0.79) a in comparison to the Framingham Risk Score (AUC = 0.61) or three CT-based statistical scores (AUCs 0.64, 0.64, and 0.62) in a large-scale sample of roughly 10,000 subjects. Although time to event did not figure within the statistical projection of this classification task, this approach has since then been reproduced in nuclear cardiology studies with varying compositions of algorithms, additional clinical predictors, and comparative models.

Machine learning-based AI using nuclear imaging (either as structured or reconstructed imaging) and clinical data may outperform human experts for cardiovascular outcome prediction. Betancur et al. evaluated the ability of machine learning to predict major adverse cardiac events, defined as all-cause mortality, nonfatal myocardial infarction, unstable angina, or late coronary revascularization during 3-year follow-up of 2619 patients. The machine learning model documented a significant improvement in predictive accuracy for MACE when compared with expert readers (AUC 0.81 vs. 0.65) with a risk reclassification of 26% [14]. In another study, gradient tree boosting machine learning was used to integrate 18 clinical, 9 stress test, and 28 imaging variables from 1980 patients from the multi-center REFINE SPECT (Registry of Fast Myocardial Perfusion Imaging with Next Generation SPECT) registry that showed an AUC of 0.79 (0.77, 0.80), surpassing that of regional stress TPD 0.71 (0.70, 0.73) or ischemic TPD 0.72 (0.71, 0.74) in predicting per-vessel chance of early coronary revascularization [15].

Deep learning has been used to analyze perfusion, wall motion, and wall thickening polar maps in the same registry coupled with age, gender, end-diastolic volume, and end-systolic volumes aiming to identify per-patient and per-vessel CAD probability. This model was then externally validated in 555 patients and showed an improved AUC 0.80 (0.76–0.84) outperforming stress TPD 0.73 (0.69–0.77), and reader diagnosis 0.65 (0.61–0.69) in the per-patient and per-vessel analyses. Additionally, attention maps were generated for clinical implementation and correlated strongly with perfusion defect areas (Pearson’s *R* 0.82, *p* < 0.001) [16••]. Thereon, the extended registry (20,401 patients) was analyzed through deep learning in order to identify cardiovascular outcomes during a median follow-up of 4.4 years. Deep learning was able to better identify patients with an adverse event beyond stress and ischemic TPD (AUC 0.75 vs. 0.70 vs. 0.68) [17]; although this data is preliminary, the report also refers the use of attention maps in order to inform the user on the areas related to the classification output. Time-to-event, however, was not modeled in these studies.

Implementing AI in clinical datasets can be prospectively challenging if vast data collection infrastructures are lacking or if multiple variables need to be manually inputted in a structured manner. A recent study explored the use of gradient boosting models (XGBoost) with the least amount possible of myocardial perfusion imaging variables and manually inputted variables yet maintaining overall MACE predictive performance over expert reader and traditional risk estimation models using the aforementioned REFINE SPECT registry (*n* = 20,414). A model including all 40 available variables (ML-ALL) achieved highest prognostic accuracy in internal validation with AUC 0.798 compared to expert reader AUC 0.680 and stress TPD AUC 0.698. Notably, model reduction to include only imaging variables decreased performance with an AUC of 0.755 still overshadowing the comparators [18•].

On the PET myocardial perfusion imaging front, transfer deep learning and data augmentation have been used in a repurposed ResNet50 architecture to show how quantitative PET myocardial perfusion polar maps can predict MACE at 2 years follow-up in a sample of 1185 patients. Notably, the discriminatory capacity of the deep learning model (which did not include clinical or functional variables as in alternative studies) even surpassed non-deep learning approaches integrating clinical variables, ventricular function, and absolute perfusion quantification (AUC = 0.90 vs. AUC = 0.85 *p* < 0.05) [11]. Of note, punctual prevalence of events was marked at the end of the follow-up once again negating the time-dependence of conventional survival analysis methods. Table 2 shows a summary of relevant studies in risk prediction in nuclear cardiology through machine learning-based AI.

**Table 2:** Relevant study characteristics of reports on CAD and MACE risk prediction through machine learning-based AI

**Authors**	**Publication time**	**Journal**	**Total *N***	**Sex (female)**	**Hypertension**	**Smoking**	**Dsylipdemia**
Juarez-Orozco et al.	2018–05	Journal of Nuclear Cardiology	1234	56	55	16	34
Betancur et al.	2018–07	JACC: Cardiovascular Imaging	2619	52	65	5	57
Commandeur et al.	2019–11	Radiology: Artificial Intelligence	850	41	57	7	66
Juarez-Orozco et al	2020–01	JACC: Cardiovascular Imaging	1185	57	55	16	34
Hu et al.	2020–05	European Heart Journal—Cardiovascular Imaging	1980	34	68	25	62
Kwan et al.	2020–09	European Radiology	352	22	65	16	56
Tamarappoo et al.	2021–02	Atherosclerosis	1069	48	55	7	67
Eisenberg et al.	2021–05	Journal of Nuclear Cardiology	2079	34	68	25	62
Benjamins et al.	2021–07	International Journal of Cardiology	830	55	64	23	74
Otaki et al.	2021–07	JACC: Cardiovascular Imaging	3578	37	66	20	59
Rios et al.	2021–07	Cardiovascular Research	23,398	43	63	19	63

**Diabetes**	**Pre-test probability of CAD**	**Training *N*:test *N***	**ML method**	**ML variable modalities**	**Comparative models**	**Predicted outcome**	**Evaluation metric**
13	Intermediate-high	1:20 (1174:60)	LogitBoost	Cardiac PET variables, clinical data	ESC guideline model, SCORE risk assessment	Myocardial ischemia	AUC 0.72 vs. 0.61
						Elevated risk of MACE	AUC 0.71 vs. 0.64
26	Intermediate-high	No test	LogitBoost	Cardiac SPECT variables, clinical data	TPD, clinical expert interpretation	MACE risk	AUC 0.81 vs. 0.73
10	Na	1:3 (236:614)	Deep learning (CNN)	Cardiac CT images	Expert manual measurement of EAT	Expert manual measurement of EAT	R = 0.905
13	Intermediate-high	No test	Deep learning (ResNet50 CNN)	PET polar map images	Clinical model, ventricular function model, absolute perfusion model	MACE during follow-up (13mo, 2–28mo)	AUC 0.90 vs. 0.78 vs. 0.74 vs. 0.85
29	Intermediate-high	No test	LogitBoost	Cardiac SPECT variables	TPD, clinical expert interpretation	Early coronary revascularization	AUC 0.81 vs. 0.71
6	Low-intermediate	No test	LogitBoost	Cardiac CT variables, clinical data	Clinical expert interpretation	Coronary revascularization	AUC 0.78 vs. 0.69
7	Intermediate	No test	XGBoost + CNN	Cardiac CT images, serum biomarkers, clinical data	Calcium score, ASCVD	MI or cardiac death risk	AUC 0.81 vs. 0.75 vs. 0.74
29	Intermediate	No test	LogitBoost	Cardiac SPECT variables, clinical data	TPD, clinical expert interpretation	CAD	AUC 0.84 vs. 0.78 vs. 0.70
						Obstructive CAD	Sensitivity 0.95 vs. 0.87 vs. 0.87
						High-risk CAD	Sensitivity 0.96 vs. 0.86 vs. 0.90
8	Low-intermediate	No test	LogitBoost	Cardiac CT variables, clinical data	Clinical expert interpretation, calcium score	PET MPI	AUC 0.91 vs. 087 vs. 0.82
						Early coronary revascularization	AUC 0.90 vs. 088 vs. 0.78
8	Intermediate	5:1 (3023:555)	Deep learning (CNN)	Cardiac SPECT images, clinical data	TPD, clinical expert interpretation	Obstructive CAD in ICA	AUC 0.80 vs. 0.73 vs. .65
26	Intermediate-high	7:1 (20,414:2984)	XGBoost	Cardiac SPECT variables, clinical data	TPD, clinical expert interpretation	MACE	AUC 0.80

The clinical risk of CAD, emerging either as a pre-test probability or as a diagnostic post-test probability, exists in close relation to the variation in clinical risk factors per patient. In fact, the most recent European Society of Cardiology guidelines on the diagnosis and management of chronic coronary syndromes [19] has evidenced the space where risk modifiers can enhance estimations of pre-test probability of CAD and deliver the so-called *clinical likelihood of CAD*. With this in mind, we more recently have reported on the repurposing of the deep learning method for the identification of common cardiovascular risk factors since the network was never subjected to these clinical traits. Interestingly, this exploratory work showed how quantitative PET images seem to harbor complex patterns which can be exploited through deep learning and are inherent to such traditional cardiovascular risk traits [20]. This supports the notion that cardiovascular risk characterization can probably benefit from broad deployment of machine learning based-AI.

Finally, emerging work is approaching the implementation of machine learning-based AI for the integration of nuclear and CT imaging data for latter prognostic modeling through traditional survival models [21]. This advances the efforts to ground AI capabilities into intuitive, interpretable, and well-known analytics accessible to all clinicians.

In summary, there is a growing body of evidence on the experimentation with and implementation of machine learning-based AI on nuclear cardiology studies both concerning SPECT and PET technology for the improvement of risk-of-disease (classification of disease) and risk-of-event (prediction of adverse events) estimations. These publications still report objective divergence in methods either utilizing statistical machine learning approaches or deep learning with widely varying architectures, in dataset sizes related to the accessibility of each technique, and in performance gains ranging anywhere from 5 to 15% in AUC values. Moreover, constitutional risk estimation from the traditional perspective of survival modeling has not been addressed and represents fertile ground for advancing the edge of machine learning-based AI in the field of nuclear cardiology.

Parallelly, it must be recognized that in spite of the aforementioned disparities, there seems to be some discrete confluence on the performances delivered by AI and further research is strongly warranted.

## Clinical Applicability

The potential benefit of machine learning-based AI for improving risk prediction in nuclear cardiac imaging seems clear when reviewing the emerging literature (vide supra) and the safe implementation of these algorithms in clinical practice is being widely discussed.

This incorporation demands heavy integration with software report tools but also robust validation in prospective cohorts. Notably, also a cultural change from clinical experts and physicians is needed to assimilate the strengths of AI rather than labeling them as threatening for their clinical domain [22].

Machine learning algorithms derive the weights and features of the decision model directly from data, without a predefined set of rules. This flexibility leads to opacity in the models’ decision process as features do not necessarily reflect human interpretable concepts. Moreover, the ever-looming risk of overfitting and bias in the training process must be recognized and mitigated. Unchecked, such risk could lead to unexpected performance deviations when the algorithms are exposed to new datasets even if discrepancies in distributions with respect to training data seem minor to human interpreters.

In order facilitate safe clinical implementation, it is important to ensure that (a) representative training data is used, (b) adequate oversight/quality control of the algorithms is in place, and (c) a degree of interpretability is implemented in the models to understand the decisions process (see underside checkpoints in the “Graphical Abstract” section).

### Representative Data

In order to safeguard ensure generalizability (i.e., the capacity to apply a model to new data and achieve comparable performance, which suggests that the learned features are relevant and universal for the task at hand) in clinical practice, training on broad real-world data is essential. Presently, a variety of hardware vendors, tracers, acquisition protocols, and alignment methods are used in cardiac nuclear imaging. Yet, most datasets available for training have originated from trails or study protocols with a high degree of homogeneity. These datasets therefore might not reflect the full data variety in imaging and may prove biased towards images of certain quality standards. A timely argument around this underlying risk comes from the recent work by DeGrave et al. [23]. They showed that many algorithms trained to classify COVID-19 from chest X-rays did not in fact learn disease patterns within the image. Instead, they learned to recognize the difference in acquisition position between the COVID-19 and non-COVID cases in the training data as chest films were mostly made in a seated position in the COVID-19 cases at the emergency room instead of in conventional acquisition positions.

Careful organization of training-dataset to reflect variations in clinical practice is therefore essential. Besides this database curation, image pre-processing techniques can be used to simulate variation in image-acquisition (such as adding noise or generating variations in pixel-value distributions of the image data) [24].

### Quality Control

In myocardial perfusion evaluation, analysts consciously and unconsciously perform quality control on both the acquired data and analysis output. Quality of the images, scatter, and alignment between the tracer signal and myocardial segmentation are all scrutinized. In automated analysis using machine learning-based AI, similar quality control (QC) is paramount. In consequence, attention has been recently focused on implementing QC steps into AI analysis frameworks. Image quality is a topic affecting all areas of medical imaging. With respect to cardiac imaging, image QC steps have been implemented for cardiac MRI analysis [25, 26] as well as echocardiography [27] and brain PET imaging [28]. For PET/CT specifically, registration steps proposed for alignment of PET and CT images [29] could also be exploited to provide a QC step for (mis)alignment.

Output data can also be scrutinized against known clinical priors. For example, ventricular shape/morphology [30, 31] and myocardial and ventricular volume dynamics over the cardiac cycle can be assessed for physiological conceivability [26]. Such steps aid to flag potential weaknesses or errors in the analysis for clinician review. Moreover, they could also be used to inform further training of the algorithm using newly analyzed data to boost its performance [32].

### Interpretability

The features that machine learning-based AI uses in decision-making are self-taught and can be made up out of a complex set of neural activations (in deep learning) that are not readily translated to concepts recognizable to humans. However, understanding the motivation/decision process in algorithms may provide better insight in their clinical validity. Interpretability could additionally support discovery of previously unknown associations and concepts within the data that determine outcome. Although it is impossible to fully comprehend the algorithm’s decision process, several methods have been developed to gain insight in an algorithm’s decisions.

Attention/activation maps are illustrations that demonstrate what part of the image contributed mostly to the decision. In myocardial perfusion imaging through SPECT, such maps can be used to correlate the classification of perfusion defects to expected coronary anatomy [16••]*.* Another approach is to embed known clinical concepts into deep learning models. In previous study, we have proposed a method that ensures clinical concepts (in this case ejection fraction or septal flash) are encoded into the latent space of a variational auto-encoder, a dimensionality reduction algorithm frequently used for classification [33]. This approach allows to solve the classification process while simultaneously interrogating the importance of known clinical features in the decision process.

In its traditional approach, the weight of a deep learning model is assigned during training as point estimates (single values are assigned for each weight). Predictions made by the model are therefore fixed maximum likelihood predictions, and do not include information about the certainty or uncertainty of the output. Several methods have been proposed to incorporate confidence estimates into DL algorithms. Monte Carlo drop-out is an approach in which random weights of a conventional neural network are temporarily disabled at test-time. This results in variations in the model predictions that can be used to classify the confidence (i.e., robustness) of these predictions. Additionally, novel Bayesian neural networks bring the possibility to quantify the uncertainty of the network allowing more comprehensive output interpretation by physicians for considering or not model’s prediction in their clinical judgment [34]. Such networks use distributions that allow the models to generalize better over new datasets. As randomness is added to the weights during training, overfitting of the model to the training-data is reduced. This makes them suitable for applications in cardiac imaging, where variations in image-data (vendors and institutional differences in acquisition protocols) are common.

## Applicability Roadmap and International Statements

Very recently, efforts have been placed into bringing standardization and quality to the experimentation and application of machine learning-based AI in cardiovascular imaging. The Proposed Requirements for Cardiovascular Imaging-Related Machine Learning Evaluation (PRIME) checklist is one such example. Released in 2020, this tool outlines a comprehensive set of seven groups of crucial aspects in the development and reporting of ML models within this and arguably other medical sciences settings [35]. It provides ground for promoting uniform and nuanced reporting of ML studies while safeguarding for algorithmic errors and biases derived from misinterpretation of ambiguity within a novel area that has advanced at an unparalleled pace. Furthermore, the European Association of Cardiovascular Imaging (EACVI) and European Association of Nuclear Medicine (EANM) have recently produced a position paper on the application of AI in multimodality cardiovascular imaging comprising hybrid SPECT and PET capabilities [3]. This document shows clear parallels on general opportunities and visions for the inclusion of AI as a powerful tool in data analysis and simultaneous attention to challenges posed by this potential new standard in data analysis for instance, data harmonization, automation, clinical support, and responsibility as well as ethical principles underlining bias prevention and control.

## Future Perspectives

Today, the full-workflow of the nuclear cardiology could be assisted by AI from imaging acquisition, to automatic report generation from robust risk-of-disease (diagnostic level) and risk-of-events (prognostic level) predictions. Examples of comprehensive frameworks that include robust quality-control and monitoring processes have been proposed for cardiac imaging and will be crucial for seamless clinical integration [26, 36].

Indeed, regulatory bodies both in the USA (*see FDA. Proposed Regulatory Framework for Modifications to Artificial Intelligence/Machine Learning (AI/ML)-Based Software as a Medical Device (SaMD)*) and Europe (*see Dept. of Health and Social Care of the United Kingdom. A guide to good practice for digital and data-driven health technologies*) [3] are beginning to formalize criteria for approval of machine learning-based AI implementations, categorizing them as software as a medical device.

As AI is implemented, it seems likely that the traditional validation methods might need to be updated to include on-going training of algorithms (such as reinforcement learning) that constantly evaluate and improve the predictions based on the data available to the networks [37]. Moreover, Bayesian methods are likely to allow better informed decision-making based on the confidence levels of the algorithm’s predictions. Human readers will find themselves at the epicenter of analysis loop in order to ensure expert evaluation of cases with abnormal nuclear imaging or those with potential errors in the analysis. With the introduction of AI, faster scans at lower radiation dosages could become the new standard. Together with automated analysis, the resulting reduction in costs and time-intensiveness could potentially see nuclear cardiology evolve from a diagnostic tool alone to a method for serial follow-up to evaluate and guide patient-specific treatments.

## Conclusions

Machine learning-based AI offers the possibility to improve risk evaluation in cardiovascular disease through its implementation on cardiac nuclear studies. Current literature attest to the encouraging results obtained from applying statistical machine learning and deep learning algorithms to integrate and select numerous clinical and imaging-derived variables, to automate detection and segmentation, and to directly analyze myocardial perfusion imaging from polar maps with respect to the individual patient’s risk-of-disease (diagnostic probability) and risk-of events (prognostic estimation) in CAD. Although large methodological heterogeneity is patent in the current literature, this is a discrete sense of convergence in optimized performance through machine learning-based AI as compared to traditional evaluation methods (semi-quantitative, quantitative, and expert-based). Prospects in this field should incorporate regard for data quality, algorithm oversight, and model interpretability.
